# Stability of Transmembrane Amyloid *β*-Peptide and Membrane Integrity Tested by Molecular Modeling of Site-Specific A*β*_42_ Mutations

**DOI:** 10.1371/journal.pone.0078399

**Published:** 2013-11-07

**Authors:** Chetan Poojari, Birgit Strodel

**Affiliations:** 1 Institute of Complex Systems: Structural Biochemistry, Forschungszentrum Jülich GmbH, Jülich, Germany; 2 Institute of Theoretical and Computational Chemistry, Heinrich Heine University Düsseldorf, Düsseldorf, Germany; Consiglio Nazionale delle Ricerche, Italy

## Abstract

Interactions of the amyloid *β*-protein (A*β*) with neuronal cell membranes, leading to the disruption of membrane integrity, are considered to play a key role in the development of Alzheimer’s disease. Natural mutations in A*β*_42_, such as the Arctic mutation (E22G) have been shown to increase A*β*_42_ aggregation and neurotoxicity, leading to the early-onset of Alzheimer’s disease. A correlation between the propensity of A*β*_42_ to form protofibrils and its effect on neuronal dysfunction and degeneration has been established. Using rational mutagenesis of the A*β*_42_ peptide it was further revealed that the aggregation of different A*β*_42_ mutants in lipid membranes results in a variety of polymorphic aggregates in a mutation dependent manner. The mutant peptides also have a variable ability to disrupt bilayer integrity. To further test the connection between A*β*_42_ mutation and peptide–membrane interactions, we perform molecular dynamics simulations of membrane-inserted A*β*_42_ variants (wild-type and E22G, D23G, E22G/D23G, K16M/K28M and K16M/E22G/D23G/K28M mutants) as *β*-sheet monomers and tetramers. The effects of charged residues on transmembrane A*β*_42_ stability and membrane integrity are analyzed at atomistic level. We observe an increased stability for the E22G A*β*_42_ peptide and a decreased stability for D23G compared to wild-type A*β*_42_, while D23G has the largest membrane-disruptive effect. These results support the experimental observation that the altered toxicity arising from mutations in A*β* is not only a result of the altered aggregation propensity, but also originates from modified A*β* interactions with neuronal membranes.

## Introduction

Alzheimer's disease (AD) is the most common form of late-onset dementia resulting in the progressive impairment of memory and executive function [1]. It is associated with synaptic loss, abnormalities in neuronal function, an increase in neuronal cell death, and the extracellular accumulation of senile plaques composed of the amyloid *β*-peptide (A*β*) [2], [3]. In general, A*β* is a normal product of cellular metabolism throughout life and circulates as a soluble peptide in biological fluids. It is produced through posttranslational processing of the amyloid precursor protein (APP), a type-1 membrane integral glycoprotein via sequential cleavage by *β*- and *γ*-secretases [4]. Of the proteolytic cleavage products, which typically contain 39 to 43 residues, A*β*_42_ is recognized as the most important alloform based on its ability to elicit neurotoxicity. It is the most prevalent alloform found in amyloid plaques, and has the highest propensity to aggregate into fibrils and plaques [5], [6]. The ‘amyloid cascade hypothesis’ proposes that assemblies of A*β* initiate a process leading to neuronal dysfunction and cell death [7]. The most potent neurotoxic assemblies appear to be oligomeric, rather than fibrillar, in nature [8], [9]. There is acceptable evidence suggesting that A*β* exerts its cytotoxic effect by interacting with membranes of neurons and other cerebral cells, such as astrocytes, microglial and cerebral endothelial cells [10], [11]. A potential pathway for A*β* toxicity lies in its ability to alter biophysical membrane properties [12]–[14], causing membrane disruption and permeability [15]–[17] and thereby allowing the leakage of ions, particularly calcium ions [17]–[20].

Familial forms of AD increase A*β* production or the propensity of A*β* to aggregate [7]. Until now four genes affecting APP, presenilin-1 (PS-1), presenilin-2 (PS-2) and apolipoprotein E have been identified to be linked to AD. So far 19 pathogenic missense mutations have been discovered in APP, of which seven are located in the region encoding A*β*. English (H6R) [21] and Tottori (D7N) [22] mutants show increased fibril elongation than wild-type (WT) A*β* [23]. The Dutch mutant (E22Q) [24], [25] favors A*β* production and leads to a *β*-sheet structure [26]–[29]. The Flemish mutant (A21G) [30] forms stable oligomers with decreased fibril formation [31], while the Arctic mutation (E22G) [32] increases neurotoxic protofibril production [33], [34]. The Italian mutant (E22K) promotes faster aggregation of A*β*_40_ and A*β*_42_ [29] and the Iowa mutant (D23N) [35] forms fibrils faster than WT A*β*.

In a recent study, intact lipid bilayers were exposed to predominantly monomeric preparations of WT or different mutant forms of A*β*_40_, and atomic force microscopy (AFM) was used to monitor aggregate formation and morphology as well as bilayer integrity over a 12 hour period [36]. The goal of this study was to determine how point mutations in A*β*, which alter peptide charge and hydrophobic character, influence interactions between A*β* and the lipid surface. The Arctic, Italian, Iowa and Flemish mutations were considered. While fibril morphology did not appear to be significantly altered when mutants were prepared similarly and incubated under free solution conditions, aggregation in the lipid membranes resulted in a variety of polymorphic aggregates in a mutation dependent manner. It was further found that the ability of A*β* to disrupt the structural integrity of bilayers is notably modulated by these mutations. An enhanced bilayer disruption was reported for the Arctic mutation. It was speculated that, in comparison to WT A*β*, the increased hydrophobic nature of E22G A*β* increases its bilayer insertion. The membrane-bound oligomers of the Iowa mutation were extremely stable and the bilayer developed small, discrete areas of disrupted lipid morphology. Based on overall electrostatic and hydrophobic properties of D23N A*β* this finding could not be explained [36].

One of the aims of the current molecular simulation study is to provide a better understanding of the experimental findings provided in [36]. In general, theoretical approaches are a complement to experimental studies probing the connection between A*β*_42_ mutations, aggregation [37], [38] and A*β*–membrane interactions [39], [40]. So far various computational studies of A*β* interacting with lipids have been performed to gain structural information at an atomistic level [41]–[62]. Structural models and experimental evidence support to the claim that transmembrane A*β* is an assembly of loosely associated mobile *β*-sheet subunits [41]–[43], [48]. In a recent study, Nussinov and co-workers used molecular simulations to demonstrate that amino acid substitutions help to infer which residues are essential for A*β* channel structures [46]. The current study builds on our previous work, where we reported on the effects of lipid type and peptide oligomerization on membrane-bound WT A*β*_42_ using molecular dynamics (MD) simulations on the sub-microsecond timescale [49]. We considered helical and *β*-sheet conformations embedded in zwitterionic palmitoyl-oleoyl phosphatidylcholine (POPC) and dipalmitoyl phosphatidylcholine (DPPC), and anionic palmitoyl-oleoyl phosphatidylglycerol (POPG) lipid bilayers. We observed that POPC increases the stability of transmembrane A*β*_42_. Hydrophobic mismatch and lipid order of DPPC, and anionic surface charges of POPG bilayers are responsible for structural instabilities of A*β*_42_ in these bilayers. From the considered structures the *β*-sheet tetramer was found to be most stable as a result of interpeptide interactions [48]. We performed a quantitative analysis of the translocation of water in the A*β*_42_ -bilayer systems. We observed that this process is generally fast (within a few nanoseconds) yet generally slower than in the absence of A*β*_42_ in the bilayers. The rate limiting step is the permeation across the hydrophobic core, where interactions between A*β*_42_ and permeating H_2_O molecules slow the translocation process. Finally, we showed that the *β*-sheet tetramer allows more water molecules to pass through the bilayer compared to monomeric A*β*_42_ [49].

The goal of the present study is to investigate the effects of the charged residues K16, E22, D23 and K28 on the stability of transmembrane A*β*_42_ in a POPC bilayer and their role on membrane integrity. To this end, we perform mutational studies for monomeric and tetrameric *β*-sheet structures of A*β*_42_. We choose A*β*_42_ to be in the *β* state because there is mounting evidence that amyloid oligomers adopt a *β* conformation in the membrane [16], [63]–[66]. Circular dichroism (CD) spectroscopy indicated that A*β*_42_ when incorporated in a lipid bilayer adopts more *β*-sheet structure in comparison to the associated peptide, which is largely unstructured [16]. Furthermore, it was demonstrated that A*β*_42_ incorporation into lipid bilayers causes membrane destabilization by increasing membrane fluidity [16]. Studies on A*β*_40_ fused into a POPC bilayer argue for damage of bilayer integrity caused by small A*β* assemblies with a large proportion of *β*-sheet structure, which are embedded in the lipid bilayer [63]. Using attenuated total reflection Fourier transform infrared (ATR-FTIR) spectroscopy, de Planque *et al.* were able to conclude that the channel-like bahavior of A*β* is not caused by helical bundles of transmembrane A*β* peptides [63]. Another study employing thioflavin T fluorescence and CD spectroscopy to characterize A*β* membrane binding and permeabilization revealed that membrane leakage is directly correlated to A*β* oligomerization and *β*-sheet formation [64]. In contrast, membrane-bound *α*-helical A*β*_40_, which is only observed at high lipid-to-peptide ratios, has a low tendency to aggregate and causes only minor membrane leakage [64]. Other recent studies show that it seems to be a generic feature of amyloid proteins to permeabilize membranes when assembled into a *β*-sheet oligomer, thereby inflicting cytotoxicity [15], [66]–[68].

However, the structure of A*β* in membranes is still not known. Therefore, models based on previous experimental and simulation results have to be designed for simulation studies investigating membrane-bound A*β*. To this end, we employ a bottom-up approach using structures predicted to be favourable for the membrane-inserted monomer and small oligomers as possible subunits for larger trans-membrane A*β* aggregates [48], [49]. In the current study, we consider the Arctic mutant E22G A*β*_42_, the ‘Arctic-type’ D23G mutant [69] in order to have a direct comparison with E22G, and the double mutant E22G/D23G. For the investigation of the effects of the positive charges of K16 and K28 we mutate these residues to methionine leading to the double mutant K16M/K28M A*β*_42_. We choose methionine since, compared to all other amino acids, it has the smallest free energy barrier for translocation across the membrane headgroup region in either direction [70], [71]. Finally, we study the quadruple mutant K16M/E22G/D23G/K28M where all peptide charges in the transmembrane region are removed. Our simulation results for WT, E22G and D23G A*β*_42_ allow a better explanation of the experimental findings testing the connection between A*β* point mutations and A*β* -induced membrane disruption [36]. To our knowledge, this study is the first computational one to investigate the effects of the familial E22G mutation on A*β* –membrane interactions.

## Results and Discussion

**Figure 1:**
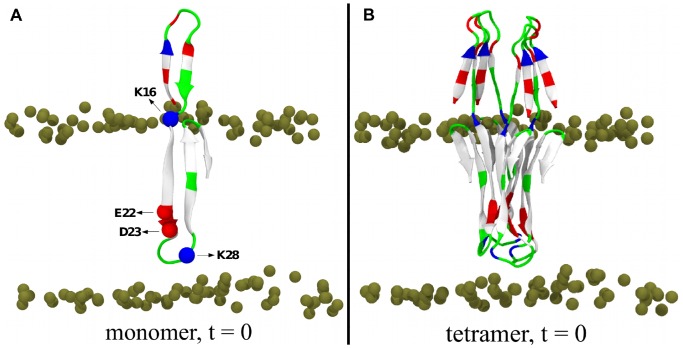
Initial structures for the MD runs. (A) *β*-sheet monomer, (B) *β*-sheet tetramer. The peptide is shown in cartoon and colored based on the physicochemical properties of the residues: blue, basic; red, acidic; white, hydrophobic; green, polar. The bilayer phosphorus atoms are shown as van der Waals spheres in tan color. Lipid tails and water molecules are not shown for clarity.

In a recent study we investigated membrane-bound WT A*β*_42_ as a *β*-sheet monomer and tetramer and as a helix [49]. The results obtained for the *β*-sheet structures will serve as comparison for the A*β*_42_ mutants considered in the current study. The monomeric and tetrameric transmembrane A*β*_42_ structures, from which our MD simulations were initiated, are shown in Fig. 1 [48].

### A*β*_42_ Monomer: Effects of Charge Removal on Transmembrane Stability

**Figure 2:**
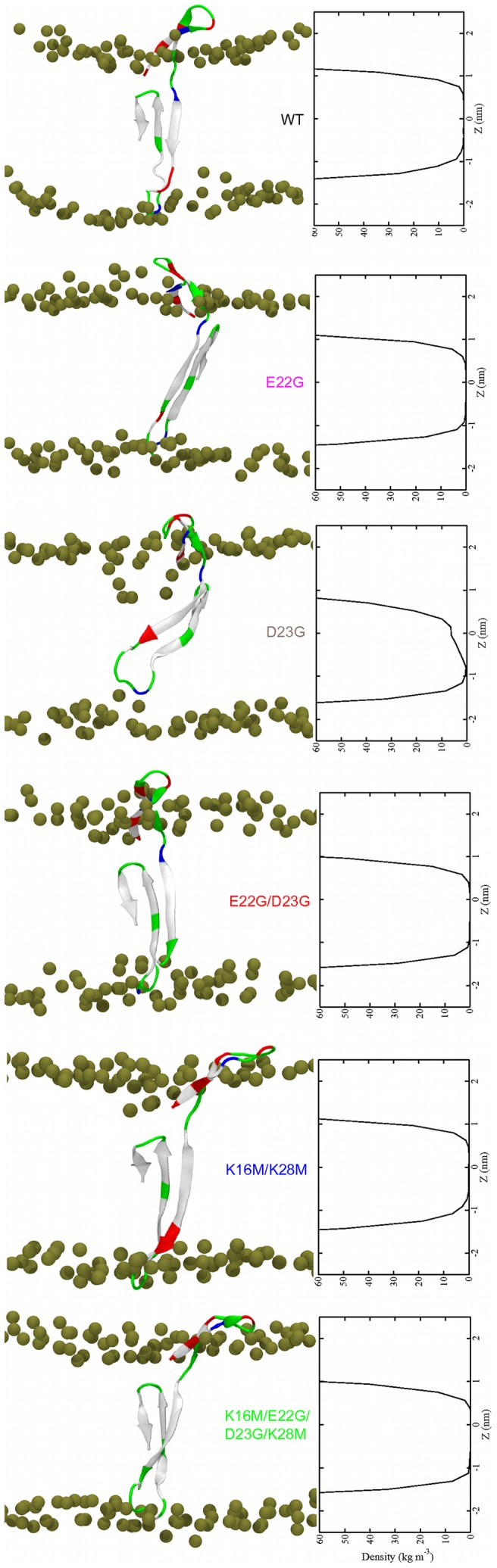
A*β*_42_ monomers in a POPC bilayer. (Left) Final structures at *t* = 500 ns. The coloring explanation for the peptides and lipids is given in Fig. 1. (Right) Time averaged water density within the bilayer.

WT and A*β*_42_ mutants were studied as monomer in the transmembrane *β*-sheet conformation in 500 ns MD simulations. The A*β*_42_ monomer structures collected at the end of the MD simulations are shown in Fig. 2.

#### Transmembrane A*β*_42_ forms stable *β*-sheets

**Figure 3:**
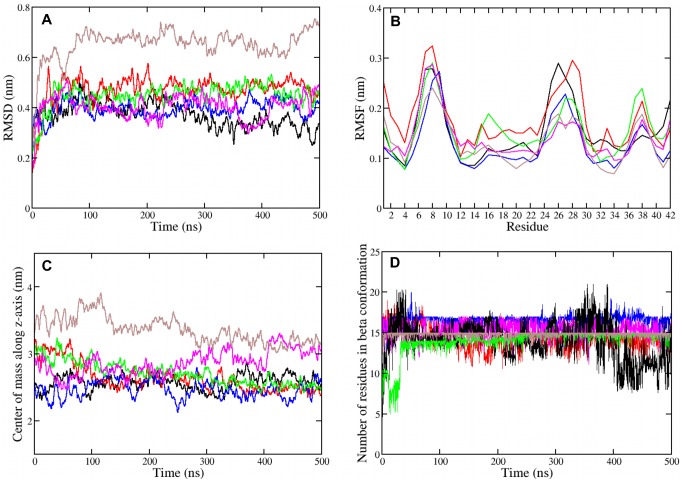
Structural analysis of A*β*_42_ monomers in a POPC bilayer. (A) Backbone root mean square deviation, (B) backbone root mean square fluctuation, (C) center of mass motion of residues 25 to 30, (D) number of transmembrane residues in *β* conformation for the *β*-sheet monomer of wild type and mutant A*β*_42_ : black, WT; magenta, E22G; ochre, D23G; red, E22G/D23G; blue, K16M/K28M; green, K16M/E22G/D23G/K28M.

Like WT A*β*_42_ [49], all of the mutants remain in the lipid bilayer during the MD simulations. In general, the root mean square deviations (RMSD) and root mean square fluctuations (RMSF) for the peptide backbone atoms, which are presented in Fig. 3A and B, reveal a similar stability and fluctuation pattern for the A*β*_42_ variants. D23G has the largest RMSD with values between 0.6 and 0.7 nm from the starting structure, while the RMSD of the other A*β*_42_ variants fluctuates around 0.4 nm. The RMSD and RMSF are not sufficient to assess the structural change and stability of transmembrane peptides. For example, both E22G and K16M/K28M have average RMSD values of 0.40 nm, while the conformations shown in Fig. 2 reveal that the structures are different inside the membrane. Therefore, we consider further observables in order to evaluate the stability of the membrane-inserted A*β*_42_ mutants. To quantify the peptide motion along the membrane normal (i.e., the *z*-direction) inside the bilayer, we compute the center of mass motion of residues 25–30 in the lower A*β*_42_ loop. The results of this analysis in Fig. 3C indicate that, after the initial 100 ns, the position of A*β*_42_ inside the membrane is stable. Only for E22G and D23G a more pronounced motion along *z* is observed leading to average positions of 2.9±0.2 nm and 3.3±0.2 nm, respectively. For the other four A*β*_42_ variants the average position inside the membrane is 2.5–2.6 nm with a standard deviation of 0.1 nm. The origin of the motion of E22G and D23G will be discussed below. Despite the removal of charged residues at the lipid–water interfaces, which in principle might act as electrostatic anchors in the transmembrane *β*-sheet structure of WT A*β*_42_, the transmembrane *β*-sheet has a high propensity to stay inside the membrane. This is attributable to the many hydrophobic amino acids between residues V24–A42, irrespective of the backbone carbonyl and amide groups which are not H-bonded in the *β*-sheet structure [49]. Between 14 and 17 out of the 26 residues between L17 and A42 are in *β*-conformation (Table 1 and Fig. 3D) with only minor differences in the average *β*-strand content and its fluctuation between the different A*β*_42_ variants. These transmembrane *β*-sheets are thus stable what is further supported by the final MD states and secondary structure analysis (Figs. S1–S3 in File S1).

**Table 1:** Peptide and POPC bilayer properties resulting from membrane-inserted WT and mutant A*β*_42_.

A*β*_42_		hyropathy	*β* count	Area per lipid [Å^2^]		bilayer	# H_2_O
mutant		index	per peptide	top	bottom	thickness [nm]	passage
	peptide-free†	n/a	n/a	69.3±0	69.3±0.0	3.51±0.0	0
monomer	WT	8.6	14±2	62.6±1.5	63.4±2.0	3.55±0.04	1
	E22G	11.7	15±1	66.1±1.3	66.3±1.2	3.54±0.05	3
	D23G	11.7	15±1	64.1±1.0	69.9±1.1	3.51±0.04	5
	E22G/D23G	14.8	14±1	66.7±1.0	66.8±0.9	3.54±0.05	2
	K16M/K28M	20.2	17+1	67.3±0.9	66.7±1.0	3.51±0.04	4
	K16M/E22G/D23G/K28M	26.4	14±2	64.8±1.1	67.2±0.8	3.55±0.05	5
tetramer	WT	8.6	15±1	65.3±1.2	62.0±1.0	3.49±0.05	5
	E22G	11.7	15±1	65.4±1.2	65.2±1.6	3.46±0.04	8
	D23G	11.7	14±1	63.2±2.0	69.1±1.4	3.48±0.05	22

Provided are the hydropathy index of WT and mutant A*β*_42_ using the hydropathy scale of Kyte and Doolittle [78], time-averaged values for the number of transmembrane residues in *β* conformation per peptide, the area per lipid headgroup in the top and bottom leaflet, the P–P bilayer thickness (each quantity with standard deviation), and the number of water molecules passing through the POPC bilayer.

† The bilayer values for the peptide-free bilayer are taken from a 100 ns MD simulation of a POPC-only bilayer [49].

It should be noted, though, that is important to consider that the force field chosen may affect the outcome of the study. The systematic evaluation of recent force fields has shown that many of them overly bias helical structures [72], [73], while the GROMOS96 53A6 parameter set [74] employed here may overstabilize extended configurations and understabilize helices [75]. However, we recently demonstrated that the GROMOS96 53A6 force field is able to reproduce the NMR shifts for A*β*_40_ and A*β*_42_, indicating that the A*β* structures it produces are in agreement with experimental observations [76]. Furthermore, the GROMOS96 53A6 protein force field is compatible with a modified version of the popular Berger force field for lipids [77]. In our previous work [49] and also in work by Lemkul and Bevan [50]–[53] this combination of protein and lipid force fields was used to study A*β*_42_ –membrane interactions. These studies revealed that A*β* in membranes is stable as both helix and *β*-sheet. The *β*-strand content is enhanced by the presence of raft membranes containing ganglioside GM1 [52] and the aggregation of A*β* inside the membrane [49], [53].

#### A*β*_42_ mutants display different transmembrane structures

The A*β*_42_ mutants exhibit somewhat higher mean RMSD values than WT A*β*_42_. While the RMSD analysis is a measure for the overall motion of the peptide residues, the RMSF highlights the flexibility of individual A*β*_42_ residues. The RMSF results in Fig. 3B indicate that the transmembrane A*β*_42_ peptide (WT and mutants) is most flexible in the three turn regions, where the first one (residues 5–11) is outside the lipid bilayer and the other two (residues D23–G29 and G37–G38, respectively) are located within the hydrophobic core. The degree of fluctuation is different for each A*β*_42_ variant and will be discussed below for each mutant. WT and E22G/D23G A*β*_42_ are most flexible between residues 22 and 30. This WT result is at first sight surprising, as it is the only monomeric *β*-sheet where this turn region is occasionally stabilized by the intramolecular D23G–K28 salt bridge (Fig. S6 in File S1). However, the breakage and formation of the salt bridge induces structural flexibility. Moreover, while the existence of the D23G–K28 salt bridge constrains the turn it does not prevent the loss of *β* structure in this region, which converts to coil-turn-coil after 400 ns of this simulation (Fig. S1 in File S1). The instability of the WT A*β*_42_ transmembrane *β*-sheet also results from residues E22 and D23 being located in the hydrophobic membrane core, where they can interact with the lower headgroups thereby destabilizing the *β*-sheet structure in this region.

#### E22G

The most stable transmembrane *β*-sheet is observed for the Arctic mutant as judged by the final MD structure (Fig. 2) and the DSSP plot (Fig. S2 in File S1). The *β*-sheet stability of E22G with 15±1 residues in *β* conformation originates from the removal of the negatively charged E22 residue, leading to overall charge neutrality inside the membrane. The mutation increases the hydropathy index from 8.6 for WT A*β*_42_ to 11.7 for E22G using the hydropathy scale of Kyte and Doolittle [78] (Table 1). The stability of E22G A*β*_42_ is further supported by the RMSF result. The larger RMSD value and motion along *z* compared to WT and most other A*β*_42_ variants result from the tilt of E22G A*β*_42_ inside the POPC bilayer. The reorientation occurs after 200 ns of the MD simulation and is only observed for this mutant. It is mainly driven by electrostatic interactions between E22G A*β*_42_ and both headgroup regions (Fig. S7 in File S1). Such a tilt is in agreement with the experimental observation that the human islet amyloid polypeptide adopts an orientation of about 48° relative to the membrane surface when interacting with a dipalmitoylphosphoglycerol (DPPG) monolayer, which might have a strong damage to the lipid membrane [66].

#### D23G

In the Arctic-type mutant [69] the salt bridge between residues D23 and K28 cannot be formed, which removes the distance constraint between these two residues and thus destabilizes the original bend region between G25 and K28 as shown in Fig. 2 and Fig. S2 in File S1. Instead, residues D23–A30 adopt mainly coil conformations, resulting in a widened loop region, which allows E22 to interact with the upper headgroups. This interaction is accompanied by an upward movement and bending of the peptide (Fig. 3C), while the lipids surrounding the peptide in the upper leaflet move downwards. This can be seen from the positions of the lipid headgroups in Fig. 2 and will be discussed in detail in the next section. The D23G mutant shows the largest deviation from the starting structure, with an average RMSD value of 0.65 nm. D23G is nonetheless stable as *β*-sheet, as confirmed by the secondary structure analysis yielding, on average, 15 residues in *β* conformation inside the membrane. The interactions between K28 with the headgroups in the lower leaflet prevent the *β*-sheet from completely moving to the upper membrane–water interface. The balance between the interactions of E22–upper headgroups and K28–lower headgroups gives rise to a stable conformation, as demonstrated by the small fluctuations according to the RMSF analysis. However, on longer time scales the D23G mutant may migrate to the upper membrane-water surface, adopting a membrane-adsorbed rather than a transmembrane conformation.

#### E22G/D23G

A stable transmembrane *β*-sheet is regained for the double mutant E22G/D23G due to the removal of both negative charges from residues 22 and 23, increasing the hydropathy index to 14.8. The elimination of the salt bridge between residues 23 and 28 leads to an extended loop region between residues 22 and 30 in E22G/D23G A*β*_42_, which induces fluctuations in both transmembrane turns and reduces the *β* count to 14±1 residues. The deletion of charged groups removes any electrostatic interactions involving residues 22 and 23 with the lower headgroup region, while the interactions between the positive charge of K28 and the lower headgroup anchors the transmembrane position of the double mutant E22G/D23G, which stabilizes this configuration.

#### K16M/K28M

From all considered mutations the K16M/K28M double mutation has the highest transmembrane *β* content with on average 17±1 residues in *β* conformation. The mutation K16M allows the peptide to move more easily along the membrane normal [70], [71], enabling the peptide to move downwards (Fig. 3C) so that the turn residues G25–N27 are exposed to the water phase and the charged residues E22 and D23 can better interact with the membrane–water interface (see final structure in Fig. 2). These interactions reduce the conformational dynamics of K16M/K28M as demonstrated by the RMSF analysis, enabling it to form a stable transmembrane structure. Furthermore, the substitution of both lysine residues with methionine raises the hydropathy index of this A*β*_42_ mutant to 20.2, causing it to be stable in a hydrophobic environment.

#### K16M/E22G/D23G/K28M

The quadruple mutant is also stable as transmembrane *β*-sheet. However, compared to the other A*β*_42_ variants it is more flexible inside the membrane. The complete removal of charged residues inside the membrane induces peptide flexibility in the upper leaflet, involving residues 15–19 and 37–38 (see RMSF analysis in Fig. 3B). Because of the missing constraint from the salt bridge between residues D23 and K28 an extended loop is formed involving residues G22–A30. The stability of this mutant as transmembrane *β*-sheet can thus be attributed to i) the inherent stability of this sheet structure, and ii) hydrophobic interactions between the peptide (its hydropathy index is 26.4) and the membrane core. Furthermore, the structure of the peptide is not perturbed by charged peptide residues positioned in the membrane core.

### A*β*_42_ Monomer: Effects of Charge Removal on Lipid Bilayer

#### Area per lipid

Table 1 summarizes the effects of the A*β*_42_ mutants on lipid bilayer properties. It shows that the insertion of A*β*_42_ into a POPC bilayer leads to a decrease in the area per lipid compared to the peptide-free bilayer. This area reduction is largest for WT A*β*_42_ with area values of 6–7 Å^2^ below the value for the pure POPC bilayer. It results from electrostatic attraction and H-bonds between A*β*_42_ residues and lipid headgroups. Removal of charged A*β*_42_ residues results in smaller reductions of the area per lipid with most values being only 2–4 Å^2^ below the area of the peptide-free POPC bilayer. Interestingly, for E22G, where peptide charges were removed in the lower leaflet, not only the area per lipid in the lower but also in the upper leaflet are larger compared to WT A*β*_42_. This result shows that the lipid packing in both leaflets is coupled to each other. For D23G, on the other hand, the area per lipid is reduced by about 5 Å^2^ in the upper leaflet and slightly increased in the lower leaflet compared to the peptide-free POPC bilayer. This behavior can be explained by the conformational instability of the D23G mutant inside the bilayer, which causes the whole peptide to move upward and bend to allow E22 to interact with the upper headgroups.

#### Bilayer thickness

This D23G–POPC interaction also leads to a marked reduction of the bilayer thickness around the peptide, which is for D23G most pronounced compared to the other mutants (Figs. S8 and S9 in File S1). For the average bilayer thicknesses we find that they are hardly affected by embedded A*β*_42_ with thickness changes within ±0.05 nm compared to the peptide-free POPC bilayer (Table 1). However, Fig. S9 in File S1 reveals that the POPC bilayer thickness around A*β*_42_ is decreased in order to improve the hydrophobic matching between bilayer and A*β*_42_, whose hydrophobic width is smaller than those of the lipids (i.e., negative hydrophobic mismatch). The POPC bilayers have a thickness of about 2.5–3.0 nm in the neighborhood of the A*β*_42_ peptides, which corresponds to the hydrophobic width of the latter for *β*-sheet structures. However, for the quadruple mutant we observe only minor changes to the bilayer thickness around the peptide, because the four mutations increase the hydrophobic width of the *β*-sheet. In general, the thinner bilayer region close to A*β*_42_ is compensated by a slight increase in thickness of the bilayer as the distance from A*β*_42_ increases [49], [51], [79]. Because of this compensation effect the average bilayer thicknesses of the A*β*_42_/POPC systems are nearly identical to the thickness of the peptide-free bilayer.

#### Lipid order

An increased bilayer thickness results from increased lipid chain order. We therefore calculated the order parameter *S_CD_* of the palmitoyl chains separately for lipids within 0.5 nm of A*β*_42_, and for the lipids, which are more than 0.5 nm away from A*β*_42_. The results of this analysis are shown in Figs. S10 and S11 in File S1. In these figures we also present *S_CD_* of the POPC-only bilayer obtained from a 100 ns MD run of the peptide-free POPC bilayer [49] for comparison. The lipid order is generally decreased around the peptide as evidenced by the *S_CD_* values, which are smaller than the one for the peptide-free bilayer, while the lipid order is marginally increased for the lipids further away from the peptide. The lipid order reduction around A*β*_42_ is strongest for E22G, D23G and K16M/K28M, while removal of all charged residues inside the membrane leads to such a good integration of the quadruple A*β*_42_ mutant into the hydrophobic membrane core that the lipid order is almost unaffected by the peptide. Especially carbon atoms 6–15 of the palmitoyl chains are not disturbed by the presence of this A*β*_42_ variant. This finding is in agreement with our observation that this particular mutant does not decrease the bilayer thickness around the peptide.

#### Water permeation

The lipid disorder around A*β*_42_ allows water molecules to enter the membrane hydrophobic core in the vicinity of the peptide. The water density profiles in Fig. 2 give an estimate for the water penetration into the membrane. The density profiles show that the headgroup regions of both bilayer leaflets are fully solvated. In all systems we see water penetration into the hydrophobic core, which is more prevalent in the top leaflet than in the bottom leaflet. This cannot be explained by the area per lipid as this quantity is generally smaller in the top than in the bottom leaflet. Yet one would expect that a large area per lipid allows water molecules to enter more easily. Instead, the interaction of the N-terminal residues 1–16 with the membrane surface, which disturbs headgroup packing (Fig. 2), facilitates the entry of water molecules into the membrane.

Lipid disorder is another factor, which could increase water penetration. The highest degree of water penetration is observed for D23G, which also shows the largest lipid disorder in the upper leaflet. Here, the water density has vanished only at *z*≈–1.0 nm, while for WT A*β*_42_ and the other mutants the water densities are zero between –1nm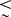 *z
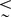
*+0.5nm. Water permeation is nonetheless small for D23G: only 5 water molecules passed through the membrane within 500 ns (Table 1). In case of the quadruple mutant we also observed 5 water translocation events, while for the other A*β*_42_ peptides this number was even smaller with values between 1 (WT A*β*_42_) and 4. For the peptide-free bilayer we did not observe any permeation event within 100 ns. We thus conclude that membrane-insertion of A*β*_42_ monomer leads to a slight increase of membrane permeability [49], which gets amplified by lipid disorder resulting from peptide–lipid interactions. However, the example of K16M/E22G/D23G/K28M shows that other factors, such as the peptide charge distribution on both sides of the membrane, also affect water permeation through the membrane, since the lipid order is least impaired by this peptide.

### A*β*_42_ Tetramer: Effect of Charge Removal on Transmembrane Stability

**Figure 4:**
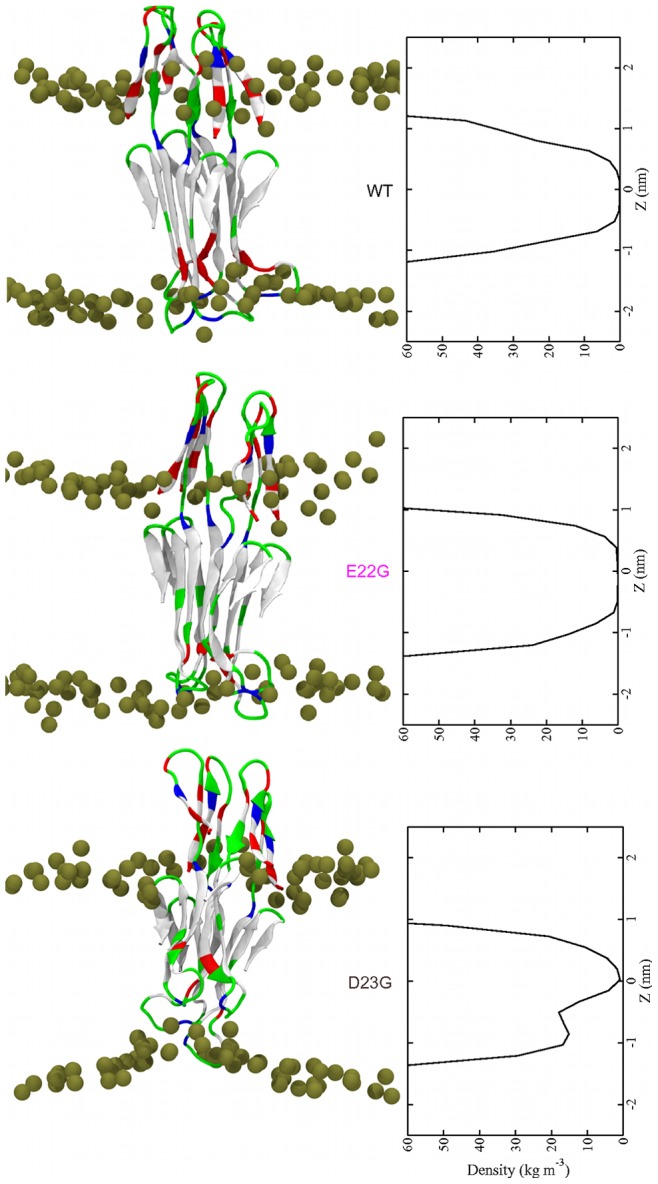
A*β*_42_ tetramers in a POPC bilayer. (Left) Final structures at *t* = 500 ns. The coloring explanation for the peptides and lipids is given in Fig. 1. (Right) Time averaged water density within the bilayer.

Our motivation for studying transmembrane *β*-sheet tetramers was to test whether they are more stable than the monomeric *β*-sheets and may constitute likely membrane-bound A*β* structures, which are able to induce membrane damage. We performed the simulations for the tetramer only for WT, E22G and D23G A*β*_42_ as these are the biologically relevant A*β*_42_ variants. The final structures of these 500 ns MD simulation are shown in Fig. 4. The results for the WT A*β*_42_ tetramer in POPC were discussed in detail in our previous study [49].

#### Oligomerization increases transmembrane stability

**Figure 5:**
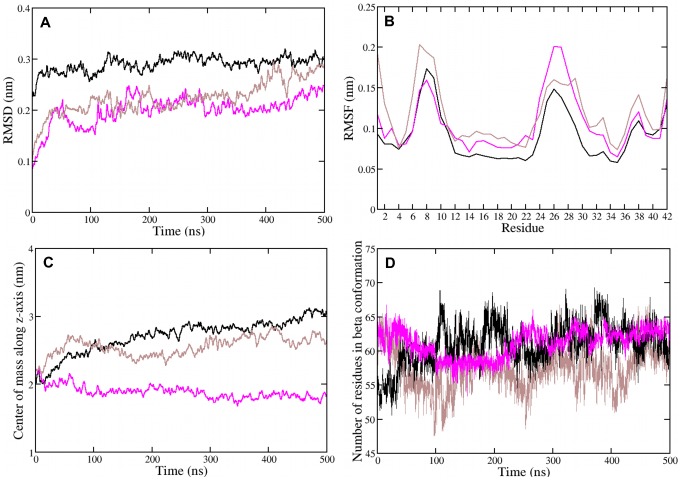
Structural analysis of A*β*_42_ tetramers in a POPC bilayer. (A) Backbone root mean square deviation, (B) backbone root mean square fluctuation, (C) center of mass motion of residues 25 to 30, (D) number of transmembrane residues in *β* conformation for the *β*-sheet tetramer of wild type and mutant A*β*_42_ : black, WT; magenta, E22G; ochre, D23G.

In all three cases we observe that, unlike in the monomeric *β*-sheets, the N-terminal *β*-hairpins are stable in the tetramer (see final snapshots and DSSP plots in Figs. S1, S4 and S5 in File S1). The *β*-hairpins interact with each other rather than with the bilayer surface, causing the N-terminal regions to protrude above the membrane instead of being adsorbed to the bilayer surface, as we observed for the *β*-sheet monomers. In larger A*β* assemblies composed of mobile *β*-sheets [43], [48] the water-exposed *β*-hairpins structure might act as a funnel for cations to be inserted into the membrane [49], [80]. The transmembrane tetramers are more stable than the *β*-sheet monomers, when the RMSD analysis in Figs. 3A and 5A are compared. The average RMSD values for the tetramers are between 0.20 and 0.30 nm, while they increased to values of 0.35–0.65 nm for the monomers. The increased stability of the tetramers can be explained by interpeptide interactions [48], [49]. The different stabilities of E22G and D23G in relation to WT A*β*_42_ tetramers will be now discussed in detail.

#### E22G

The Arctic mutant E22G is more stable than both WT and D23G A*β*_42_ tetramers as the RMSD analysis (Fig. 5A) and secondary structure plot (Fig. S4 in File S1) reveal. The *β*-sheet structure in the hydrophobic core is well conserved throughout the simulation (Fig. 5D). The number of residues in *β* conformation per peptide is very similar to the *β* content of the E22G monomer and the WT A*β*_42_ tetramer (Table 1). Interestingly, the *β* content for the E22G tetramer is lowest between 100 and 200 ns when the salt bridge D23–K28 in one of the four peptides is formed (Fig. S6 in File S1). That the salt bridge does promote *β* conformation in this region was already observed during the last 100 ns of the WT monomer simulation. In general, the lower turn region involving residues 23–29 is the most flexible part in the peptides composing the E22G tetramer. The RMSF plot (Fig. 5B) shows that these residues fluctuate more than in both WT and D23G tetramer. This can be explained with the deep insertion of the turn region into the lower headgroup region, where the peptide structure becomes disturbed by interactions with the headgroups and water molecules. The downward motion between 100 and 200 ns is revealed by the analysis of the peptides center of mass motion along *z* (Fig. 5C). Because of interpeptide interactions the *β*-sheets inside the membrane do not tilt as was observed for the E22G monomer.

#### D23G

The D23G tetramer remains stable until around 350 ns. At that time, the RMSD (Fig. 5A) increases because the peptides start bending towards the upper membrane surface. However, this bending is not as strong as in the D23G monomer as interactions between the peptides counteract the attractive forces between residue E22 and the upper headgroups. Two of the peptides of the D23G tetramer move upwards inside the bilayer, causing the surrounding lipids to move with them. This movement leads to a marked reduction of the bilayer thickness around the tetramer. The lipid headgroups of the lower leaflet are pulled upwards by interactions with with E22 and K28 in the turn region, leading to membrane disruption (discussed below). As for the D23G monomer, the absence of the salt bridge between residues 23 and 28 destabilizes the turn region thereby inducing conformational flexibility. The *β* content for the D23G tetramer belongs to the lowest values among the studied systems. While the time-averaged number of residues in *β* conformation is 15±0 for the D23G monomer, it declined to 14±1 for the tetramer. Thus, opposite to WT and E22G the oligomerization does not help stabilizing the *β*-sheet conformation in the D23G tetramer. Furthermore, the amount of *β* fluctuation has increased for the D23G tetramer. The structural flexibility is also evident in the RMSF (Fig. 5B), which is for most residues larger for D23 compared to both WT and E22G. Surprisingly, only the turn region involving residues V24–K28 fluctuates less than in the E22G tetramer despite the extended loop region in D23G A*β*_42_. This is attributable to the interaction of D23 in E22G A*β*_42_ with the lower headgroups exposing the turn regions to the water phase, which increases its flexibility.

### A*β*_42_ Tetramer: Effects on Lipid Bilayer

#### Area per lipid, bilayer thickness and lipid order

The effects on the properties of the POPC bilayer resulting from membrane-inserted WT, E22G and D23G A*β*_42_ tetramers are very similar to those observed for the corresponding monomers (Table 1). The WT tetramer has on average the largest effect on the area per lipid. E22G leads to area reductions of about 4 Å^2^ per lipid in both leaflets, while D23G leaves the area per lipid in the lower leaflet unaffected but reduces it by about 6 Å^2^ in the upper leaflet. The average bilayer thickness is 0.02–0.05 nm smaller compared to the peptide-free bilayer. This reduction results from the hydrophobic mismatch between A*β*_42_ and the membrane core, which compresses the bilayer near the peptides (Fig. S9 in File S1). This in turn is accompanied by a reduction in lipid tail order, which can be seen from the analysis of *S_CD_* of the lipids within 0.5 nm of the peptides (Fig. S12 in File S1). WT and D23G tetramers have a larger disordering effect on nearby lipids than the E22G tetramer. The latter observation differs from the finding that E22G monomer disorders neighboring lipids. None of the tetramers increased (or only marginally in the case of WT) the lipid order in the palmitoyl chains >0.5 nm away from the peptides, which explains why the average bilayer thickness is slightly reduced, and not increased as for the A*β*_42_ monomers.

#### Water permeation

The water density profiles for the A*β*_42_ tetramers (Fig. 4) reveal an almost continuous water channel in case of D23G. Only at *z*≈0 the water density has almost declined to zero. The increased membrane permeability for D23G tetramer is also demonstrated by the translocation of 22 water molecules during the simulation. This number is substantially larger than the corresponding numbers for WT and E22G tetramer (5 and 8, respectively) and for the monomers (≤5 water translocations). The increased water flow induced by the D23G tetramer is due to the greater disruption of membrane integrity especially in the lower leaflet. The water density profiles for WT and E22G tetramers also reveal an increase in the average probability of finding water inside the membrane compared to that of the monomers. Only for –0.5 nm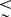 *z
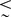
*+0.5 nm this probability is zero. Noteworthy, while the WT tetramer enables more water molecules to reside inside the membrane compared to the E22G tetramer, it supports fewer water permeation events. This again shows that membrane permeability in the vicinity of membrane-inserted amyloid peptide is a complex process, which is governed by a multitude of factors, such as lipid type, A*β*_42_ conformation (as it influences the number and strength of interactions between A*β*_42_ and permeating water molecules), and A*β*_42_ oligomerization [49]. As in our previous study [49], we observe that A*β*_42_ oligomerization is an important event, which causes an increase in membrane permeability. Here, we have shown that the removal of peptide charges inside the membrane further increases the amount of water inside the membrane and the number of permeation events.

### Conclusion

Based on the evidence that the cytotoxicity in AD originates from interactions of A*β* with neuronal cell membranes disturbing the integrity of the membrane [15]–[17], we performed mutational studies to investigate the transmembrane stability of various A*β*_42_ mutants in a *β*-sheet conformation [48]. Our 500 ns MD simulations of A*β*_1–42_ mutants in a POPC bilayer reveal a similar or increased stability compared to WT A*β*_42_ for all mutants except D23G. For the monomeric *β*-sheet we observed the highest stability for the Arctic mutant E22G and the double mutant K16M/K28M. The removal of positive charges by mutating K16 and K28 to methionine increases the hydropathy index of this mutant A*β*_42_ by a factor of 2.34, which gives rise to a stable transmembrane *β*-sheet. The stability of the Arctic mutant E22G can be attributed to the removal of the negative E22 charge in combination with D23 and K28 interacting with the headgroups of the lower leaflet, leading to charge neutrality of the peptide inside the membrane. While the ‘Arctic-type’ D23G mutant has the same hydropathy index as E22G A*β*_42_, it is not stable as transmembrane *β*-sheet, since the position of E22 inside the membrane causes the peptide to bend towards the upper membrane surface. The less toxic WT A*β*_42_, on the other hand, looses some of its *β* structure during the MD simulation due to its overall negative charge inside the membrane. For APP it was experimentally shown that the Arctic mutation alters the transmembrane localization compared to WT APP, leading to reduced levels of Arctic APP at the cell surface making it less available for non-amyloidogenic cleavage. As a result, the extent and subcellular location of A*β* formation is changed, as revealed by increased A*β* levels, especially at intracellular locations [81]. Our simulation results reveal that also for A*β* the Arctic mutation increases its propensity to remain buried inside the lipid bilayer.

In our previous study [49] we demonstrated that a single transmembrane A*β*_42_ peptide is not sufficient to explain the experimentally observed membrane damage resulting from membrane-bound A*β*, which causes cellular ionic imbalance [18]–[20]. This finding allowed us to conclude that membrane permeabilization by membrane-bound A*β* as commonly observed experimentally [15]–[17] must be due to transmembrane A*β* oligomers and not monomers as some studies conjectured [82], [83]. This conclusion is supported by the results of the current study. Based on size and biochemical considerations it is also evident that more than single A*β* peptides enter the membranes [20], [36], [84]. Therefore, the water translocation arising from a monomeric transmembrane A*β β*-sheet must not be overemphasized, yet its structural stability is a determinant for the stability of the corresponding transmembrane oligomer. For instance, the E22G A*β*_42_ mutant is very stable both as monomeric and tetrameric transmembrane *β*-sheet, while D23G is least stable in either case. On the other hand, transmembrane D23G generates the largest amount of membrane permeation compared to the other monomers and tetramers.

This computational finding can be compared to experimental observations. In situ AFM experiments with Arctic A*β*_40_ exposed to total brain lipid extract (TBLE) bilayers showed small membrane-bound oligomeric aggregates with large areas of bilayer disruption [36]. These areas were seen to be populated with stable oligomers composed of 10–15 peptides per oligomer, rather than with fibrillar aggregates as observed for WT A*β*_40_. The same kind of experiments with Iowa mutant (D23N) A*β*_40_ revealed the formation of stable oligomeric aggregates on the TBLE surface within 2–3 hours [36]. However, after longer exposure (10–12 hours), the bilayer structural integrity was highly disrupted in small areas arising from D23N A*β*_40_ oligomers inside the bilayer [36]. Our molecular simulations revealed that E22G A*β* has a higher propensity to stay inside a membrane compared to D23G, while D23G has a greater tendency to bind to the surface. However, when D23G A*β* is inside the membrane, it has a great capacity to disrupt membrane integrity. Assuming the mutation location D23 to be the crucial factor, the experimentally observed behavioral differences between membrane-bound E22G and D23N could be explained based on our simulation results. Though, given the physicochemical differences between Gly and Asn this conclusion still needs to be proven by further simulations. To our knowledge, no experiment on D23G A*β* in the presence of a lipid membrane has been carried out yet. NMR studies have revealed a large destabilizing effect of the D23G mutation on the turn region involving residues 21–30 [69], which is in agreement to our computational results of transmembrane D23G A*β*_42_.

Experimental studies of A*β* mutant peptides revealed that the aggregation propensity to form (proto)fibrils is not sufficient to explain the observed *in vivo* toxicity of the A*β*_42_ peptides [85], [86]. Our results on the interactions between A*β*_42_ and a POPC bilayer, and the effect of A*β*_42_ mutations on bilayer properties provide further insight into the likely toxicity mechanism caused by membrane-inserted A*β*_42_ oligomers. We conclude that the higher transmembrane stability of E22G and its increased membrane disturbing effect compared to WT A*β* are possible reasons for the increased cytotoxicity of Arctic A*β*. While our current simulations are still rather short investigating only small oligomers–-simulations of larger than tetrameric oligomers on the millisecond time scale would be needed, which are yet prohibitively long with respect to computing time–-we are able to state that A*β*_42_ mutations have an effect on transmembrane stability and membrane integrity. This should be motivation enough for experimentalists and simulation scientists to perform further studies on these systems.

## Methods

### Starting Structures

The initial A*β*_42_ structure is a *β*-sheet, which was obtained from a study of the A*β*_42_ monomer and small oligomers using a global optimization approach and an implicit membrane model [48]. In this structure, the more hydrophobic C-terminal region starting from residue 17 is fully inserted into the hydrophobic membrane core, forming an antiparallel *β*-sheet with two turn regions. The first turn ranges from residue 23 to 29, and the second one involves residues 37 and 38. In solution, the G37–G38 hinge structure has been identified as characteristic of A*β*_42_ distinguishing it from its C-terminal truncated relative A*β*_40_ [87]. The first turn is prominent in many A*β* structures identified from experiment [88]–[91] and simulation [92]–[94]. While each of these models predict a distinct turn structure, they share the key structural features of a salt bridge between Asp23–Lys28 and the intramolecular hydrophobic cluster between Leu17/Phe19 and Ile32/Leu34. We decided to use our *β*-hairpin model as starting structure as it also provides a structural model for the more hydrophilic residues 1–16, which form a *β*-hairpin outside the membrane [48]. The N- and C-terminals were capped to nullify the effect of terminal residues in peptide-lipid interactions. The coordinates of the monomeric and tetrameric starting structures are available from the Cambridge Cluster Database [95]. Structures obtained in the current work are available from the authors upon request.

### Molecular Dynamics Simulations

All MD simulations were performed with the GROMACS 4.0 package [96]. The A*β*_42_ peptide was described using the GROMOS96 53A6 force field [74], and the POPC lipids were modeled with modified Berger force field parameters for use with the GROMOS96 53A6 force field [77]. Initial coordinates of 128 lipids for POPC bilayer equilibrated with water for 40 ns were obtained from Kukol’s work on lipid models [77]. The A*β*_42_ peptide was inserted into the pre-equilibrated lipid membrane using the INFLATEGRO script [97]. Once A*β*_42_ was inserted into the lipid membrane, the structures were solvated with SPC water molecules, Na^+^ counterions were added to balance the peptide charge, and 0.1 M NaCl salt added to bring the system to the a physiological salt concentration. The simulations were carried out in a 6.5×6.5×9.5 nm^3^ box. An initial equilibration under isothermal-isochoric conditions was performed for 100 ps during which the protein heavy atoms and phosphorous atoms of the lipid headgroups were restrained with a force constant of 1000 kJ mol^−1^ nm^−2^. Here, a weak coupling thermostat with stochastic velocity reassignment [98] using a coupling constant of 0.1 ps was used to regulate the temperature of the peptide, lipids, and solvent/ions separately at 298 K. The systems were then equilibrated under isothermal-isobaric (NPT) conditions for 30 ns. For the NPT ensemble the Nose-Hoover thermostat [99], [100] was used to regulate the temperature along with semiisotropic Parrinello-Rahman pressure coupling [101]. The bilayer normal *z*-direction and *xy*-plane were coupled separately with a time constant of 5.0 ps maintaining a constant pressure of 1 bar independently in all directions. An isothermal compressibility of 4.5×10^5^ bar^−1^ was applied in all box dimensions. Long-range electrostatics were calculated using the Particle Mesh-Ewald method [102], [103] in connection with periodic boundary conditions. Van der Waals and Coulombic interaction cutoffs were set to 1.2 nm and the LINCS algorithm [104] was used to constrain all bond lengths. Following equilibration, production MD runs were performed for 500 ns for each system. Here the parameter settings were similar to the NPT equilibration step, except that all restraints were removed and the time constant for pressure coupling was set to 2.0 ps. The time step for integration was 2 fs with coordinates and velocities saved every 20 ps for analysis.

### Analysis

The structural stability (RMSD) and dynamic properties (RMSF) of A*β*_42_ are analyzed for backbone atoms using GROMACS tools. To characterize the effects of the peptide on the orientational mobility of the lipid molecules we calculated the lipid tail order parameter *S_CD_* defined as
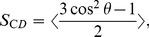
(1)where *θ* is the angle between the C–H bond vector (in the simulation) or the C–D bond vector (in the experiment) and the bilayer normal. The angular brackets indicate averaging over lipids and over time. The center of mass motion (COM) is calculated for the turn region from residue 25 to 30 of A*β*_42_ inside the membrane hydrophobic core. The secondary structure of A*β*_42_ was analyzed using the DSSP method [105] and the time-averaged values for the number of transmembrane residues (i.e., between L17 and A42) in *β* conformation per peptide computed. The salt bridge between D23 and K28 is considered to be formed when the distance between the anionic carboxylate of D23 and the cationic ammonium from K28 is <4.5 Å. We used the grid-based membrane analysis tool GRIDMAT-MD to quantify the extent to which the peptide affects the lipid headgroup arrangement and bilayer thickness [106]. For the bilayer thickness we report phosphate-to-phosphate (P–P) distances. To measure the depth of water molecule penetration into the hydrophobic core, water density profiles projected onto the *z*-direction were calculated, while water permeation across the membrane was quantified using VMD [107]. Time-averaged values were calculated for the last 400 ns of the 500 ns MD simulations.

## Supporting Information

File S1 **Contains: Figure S1** Secondary structure analysis for the 500 ns MD simulations of WT A*β*_42_ as (a) *β*-sheet monomer and (b) *β*-sheet tetramer in a POPC bilayer. **Figure S2** Secondary structure analysis for the 500 ns MD simulations of mutant A*β*_42_ monomer as (a) E22G, (b) D23G in a POPC bilayer. **Figure S3** Secondary structure analysis for the 500 ns MD simulations of mutant A*β*_42_ monomer as (a) E22G/D23G, (b) K16M/K28M, (c) K16M/E22G/D23G/K28M in a POPC bilayer. **Figure S4** Secondary structure analysis for the 500 ns MD simulation of E22G A*β*_42_ tetramer in a POPC bilayer. **Figure S5** Secondary structure analysis for the 500 ns MD simulation of D23G A*β*_42_ tetramer in a POPC bilayer. **Figure S6** Minimum distance between the anionic carboxylate of D23 and the cationic ammonium from K28 in the WT monomer and the E22G tetramer. **Figure S7** Peptide-lipid interactions for the E22G monomer decomposed into Coulomb and Lennard-Jones interactions. **Figure S8** Bilayer phosphate-to-phosphate thickness, averaged over the last 400 ns of the 500 ns MD simulations of WT A*β*_42_ as (a) *β*-sheet monomer and (b) *β*-sheet tetramer in a POPC bilayer. **Figure S9** Bilayer phosphate-to-phosphate thickness, averaged over last 400 ns of the 500 ns MD simulations of A*β* mutants (monomers and tetramers). **Figure S10** Time-averaged order parameter *S_CD_* of the palmitoyl chain of the POPC lipids. Results are shown for WT, E22G and D23G A*β*_42_ monomer. **Figure S11** Time-averaged order parameter *S_CD_* of the palmitoyl chain of the POPC lipids. Results are shown for WT, E22G/D23G, K16M/K28M and K16M/E22G/D23G/K28M A*β*_42_ monomer. **Figure S12** Time-averaged order parameter *S_CD_* of the palmitoyl chain of the POPC lipids. Results are shown for WT, E22G and D23G A*β*_42_ tetramer.
(PDF)
