# Supplementary material for: Stability of Transmembrane Amyloid β-Peptide and Membrane Integrity Tested by Molecular Modeling of Site-Specific Aβ 42 Mutations
Source: PLoS One. 2013 Nov 7;8(11):e78399. doi: 10.1371/journal.pone.0078399 (PMC3820573; doi:10.1371/journal.pone.0078399)
Supplement: File S1 — Contains: Figure S1 Secondary structure analysis for the 500 ns MD simulations of WT Aβ 42 as (a) β-sheet monomer and (b) β-sheet tetramer in a POPC bilayer. Figure S2 Secondary structure analysis for the 500 ns MD simulations of mutant Aβ 42 monomer as (a) E22G, (b) D23G in a POPC bilayer. Figure S3 Secondary structure analysis for the 500 ns MD simulations of mutant Aβ 42 monomer as (a) E22G/D23G, (b) K16M/K28M, (c) K16M/E22G/D23G/K28M in a POPC bilayer. Figure S4 Secondary structure analysis for the 500 ns MD simulation of E22G Aβ 42 tetramer in a POPC bilayer. Figure S5 Secondary structure analysis for the 500 ns MD simulation of D23G Aβ 42 tetramer in a POPC bilayer. Figure S6 Minimum distance between the anionic carboxylate of D23 and the cationic ammonium from K28 in the WT monomer and the E22G tetramer. Figure S7 Peptide-lipid interactions for the E22G monomer decomposed into Coulomb and Lennard-Jones interactions. Figure S8 Bilayer phosphate-to-phosphate thickness, averaged over the last 400 ns of the 500 ns MD simulations of WT Aβ 42 as (a) β-sheet monomer and (b) β-sheet tetramer in a POPC bilayer. Figure S9 Bilayer phosphate-to-phosphate thickness, averaged over last 400 ns of the 500 ns MD simulations of Aβ mutants (monomers and tetramers). Figure S10 Time-averaged order parameter SCD of the palmitoyl chain of the POPC lipids. Results are shown for WT, E22G and D23G Aβ 42 monomer. Figure S11 Time-averaged order parameter SCD of the palmitoyl chain of the POPC lipids. Results are shown for WT, E22G/D23G, K16M/K28M and K16M/E22G/D23G/K28M Aβ 42 monomer. Figure S12 Time-averaged order parameter SCD of the palmitoyl chain of the POPC lipids. Results are shown for WT, E22G and D23G Aβ 42 tetramer. (PDF) [file pone.0078399.s001.pdf]

Supporting Information

Stability of transmembrane amyloid β-peptide and membrane integrity tested by site-specific Aβ_{42} mutations

Chetan Poojari[1] , Birgit Strodel[1,2,*]

[1] Forschungszentrum Jülich GmbH, Institute of Complex Systems: Structural Biochemistry (ICS-6), 52425 Jülich, Germany

[2] Institute of Theoretical and Computational Chemistry, Heinrich Heine University Düsseldorf, Universitätsstr. 1, 40225 Düsseldorf, Germany

* E-mail: b.strodel@fz-juelich.de

Secondary structure of WT Aβ_{42}

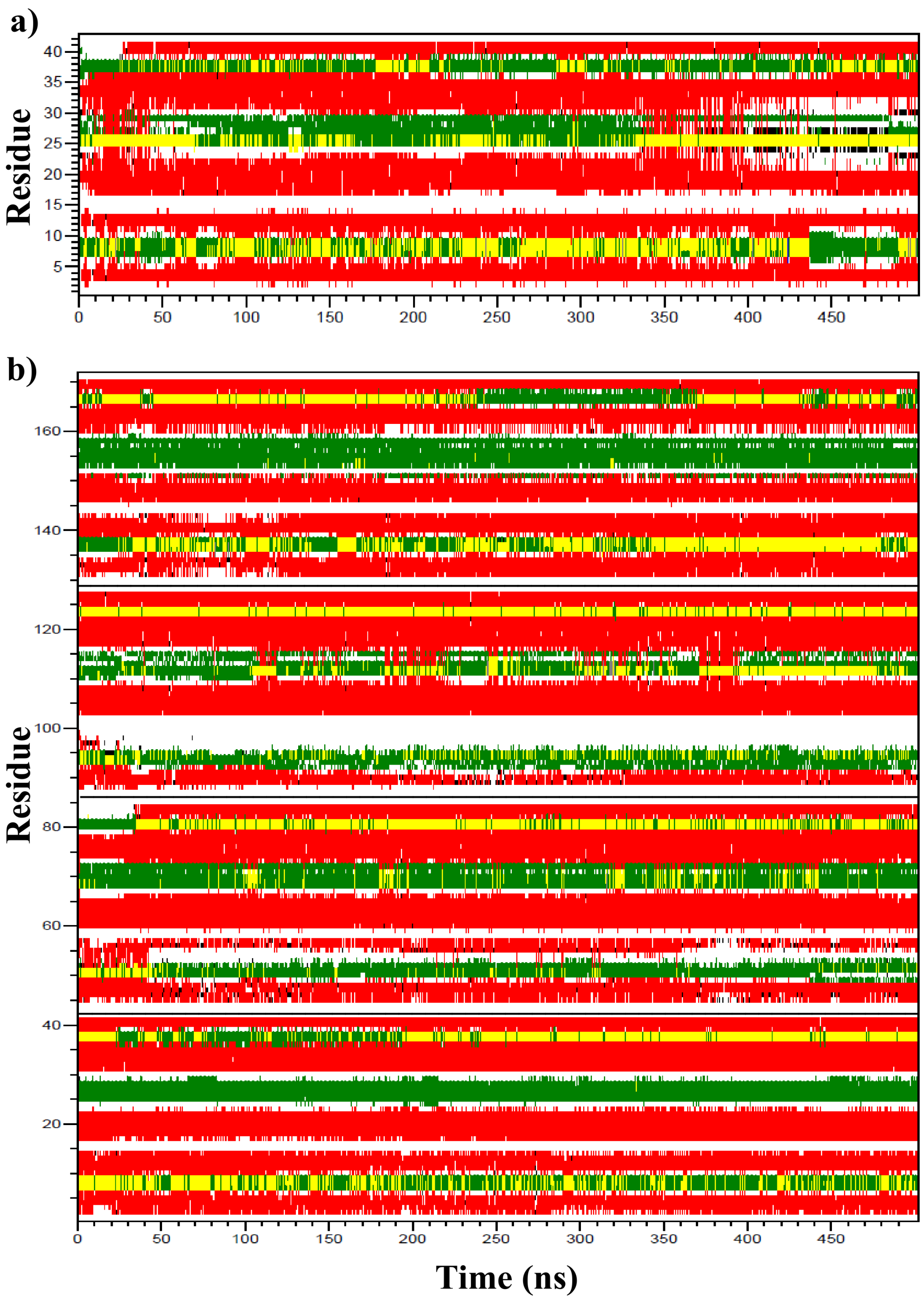

Figure S1: Secondary structure analysis for the 500 ns MD simulations of WT A$\beta_{1\text{-}42}$ as (a) β-sheet monomer and (b) β-sheet tetramer in a POPC bilayer. For the β-sheet tetramer, the four peptides are separated by black lines. Legend: white, coil; red, β-sheet; black, β-bridge; green, bend; yellow, turn.

Secondary structure of mutant Aβ$_{42}$ (monomer)

a)

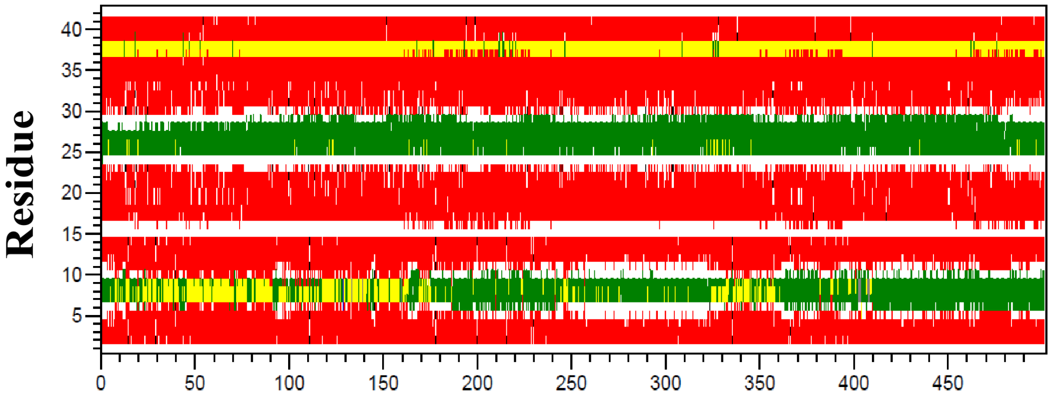

b)

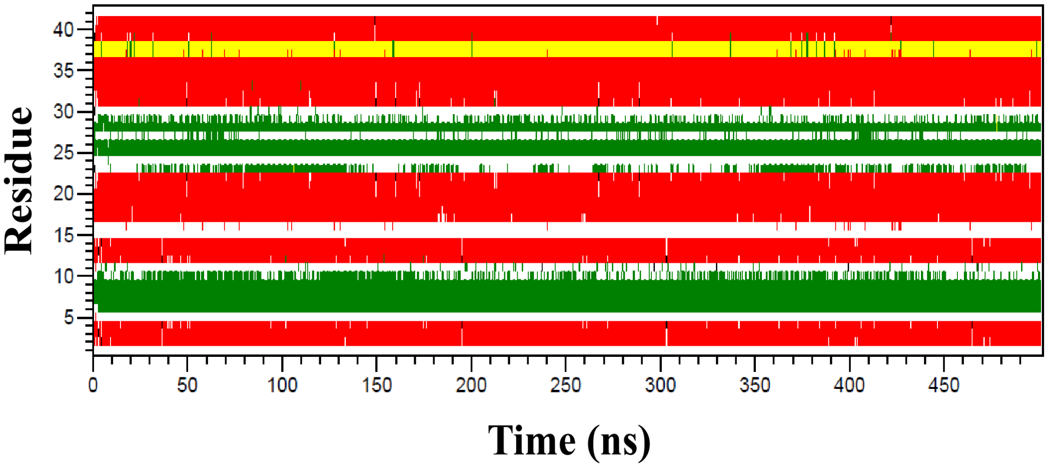

Figure S2: Secondary structure analysis for the 500 ns MD simulations of mutant Aβ$_{1-42}$ monomer as (a) E22G, (b) D23G in a POPC bilayer. Legend: white, coil; red, β-sheet; black, β-bridge; green, bend; yellow, turn.

Secondary structure for mutant Aβ_{42} (monomer)

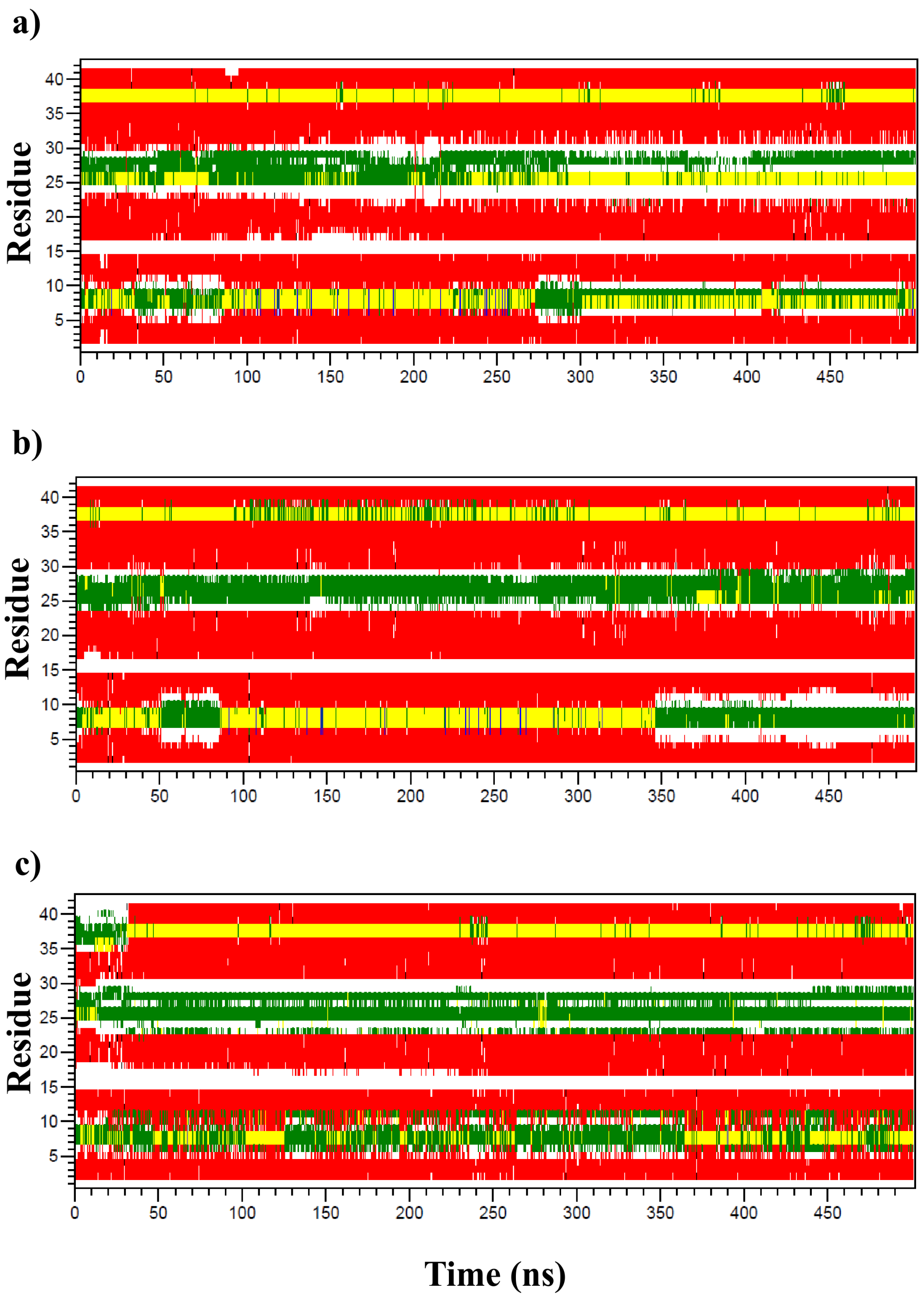

Figure S3: Secondary structure analysis for the 500 ns MD simulations of mutant A$\beta_{1\text{-}42}$ monomer as (a) E22G/D23G, (b) K16M/K28M, (c) K16M/E22G/D23G/K28M in a POPC bilayer. Legend: white, coil; red, β-sheet; black, β-bridge; green, bend; yellow, turn.

Secondary structure for E22G mutant Aβ$_{1-42}$ (tetramer)

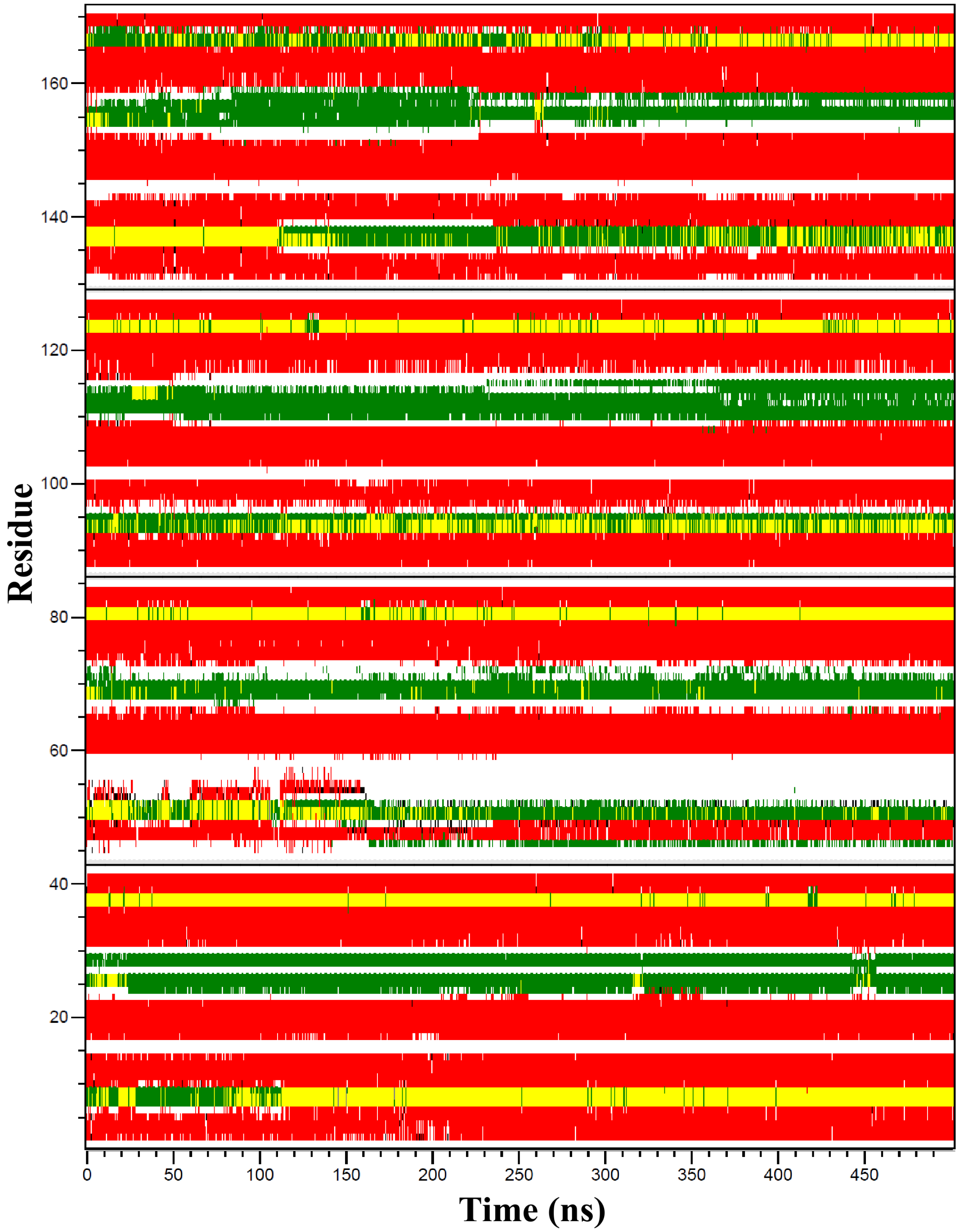

Figure S4: Secondary structure analysis for the 500 ns MD simulation of E22G Aβ$_{1-42}$ tetramer in a POPC bilayer. The four peptides are separated by black lines. Legend: white, coil; red, β-sheet; black, β-bridge; green, bend; yellow, turn.

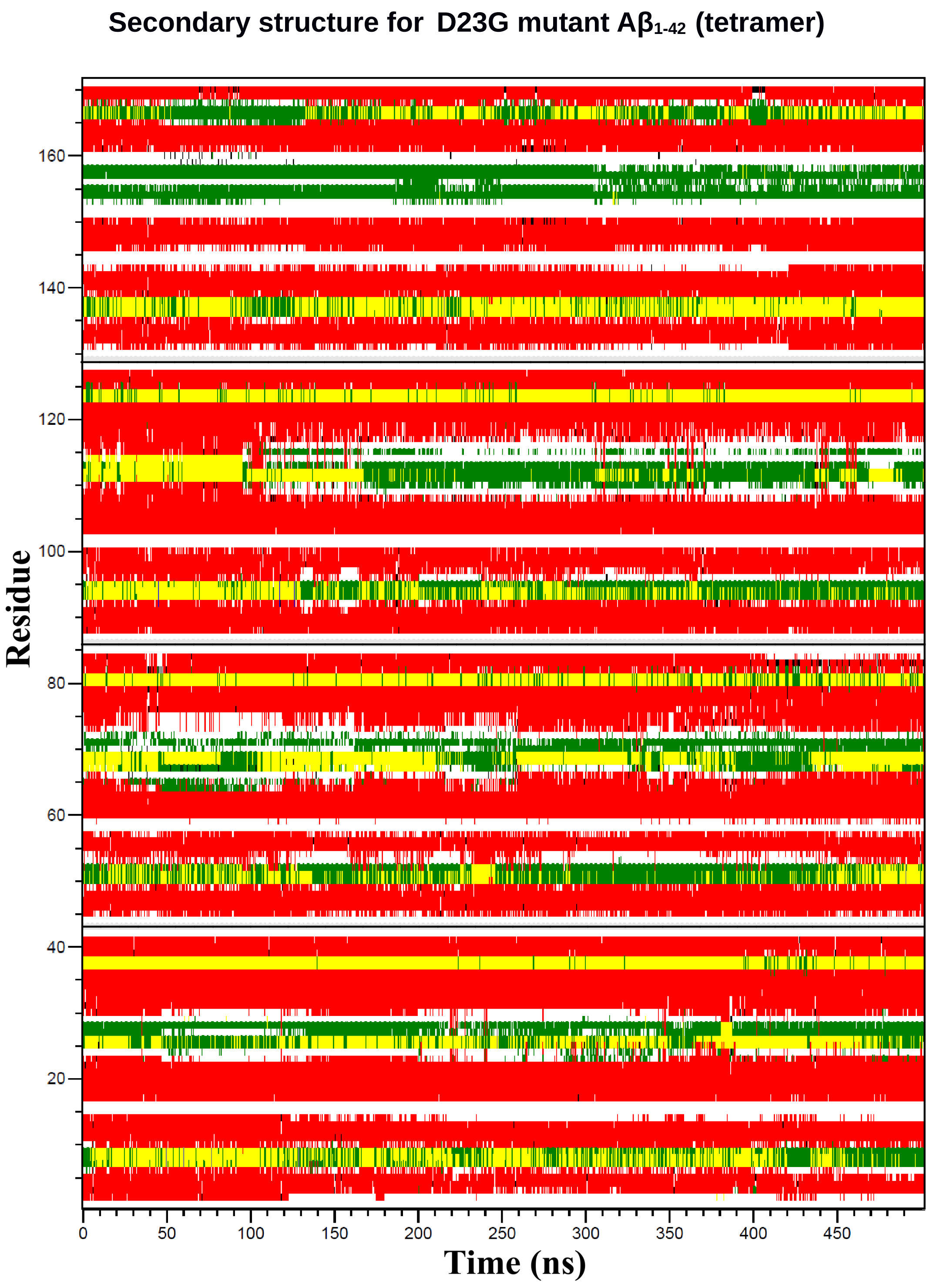

Figure S5: Secondary structure analysis for the 500 ns MD simulation of D23G A$\beta_{1\text{-}42}$ tetramer in a POPC bilayer. The four peptides are separated by black lines. Legend: white, coil; red, β-sheet; black, β-bridge; green, bend; yellow, turn.

D23-K28 salt bridge in the sheet structure

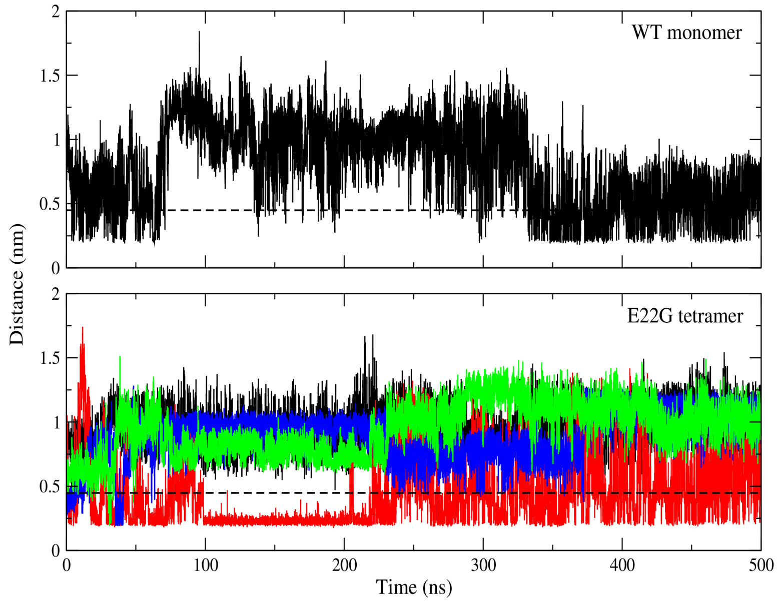

Figure S6: Minimum distance between the anionic carboxylate of D23 and the cationic ammonium from K28 in the WT monomer and the E22G tetramer. For E22G tetramer, this distance is analysed for all four peptides. The black dashed line at 0.45 nm corresponds to the cutoff distance to define a salt bridge in a protein. This analysis was performed for all systems studied. Only for the two systems, where salt bridge formation was observed, results are shown here.

Peptide-lipid interactions for E22G monomer

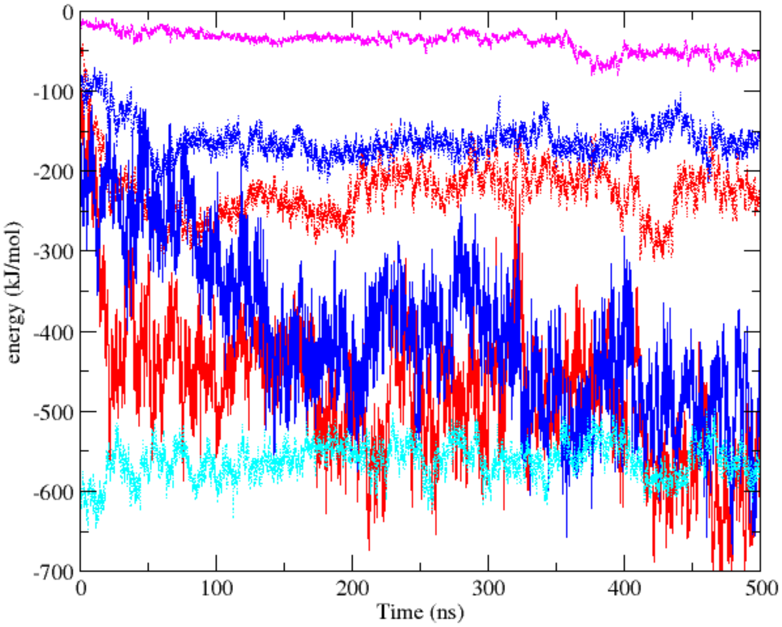

Figure S7: Peptide-lipid interactions for the E22G monomer decomposed into Coulomb (solid) and Lennard-Jones (LJ) interactions (dashed). Shown are the interactions of residues 1–16 with the lipid headgroups (red, Coulomb and LJ) and lipid tails (magenta, LJ), and of residues 17–42 with the lipid headgroups (blue, Coulomb and LJ) and lipid tails (cyan, LJ).

Analysis of bilayer thickness for wild type Aβ$_{1-42}$

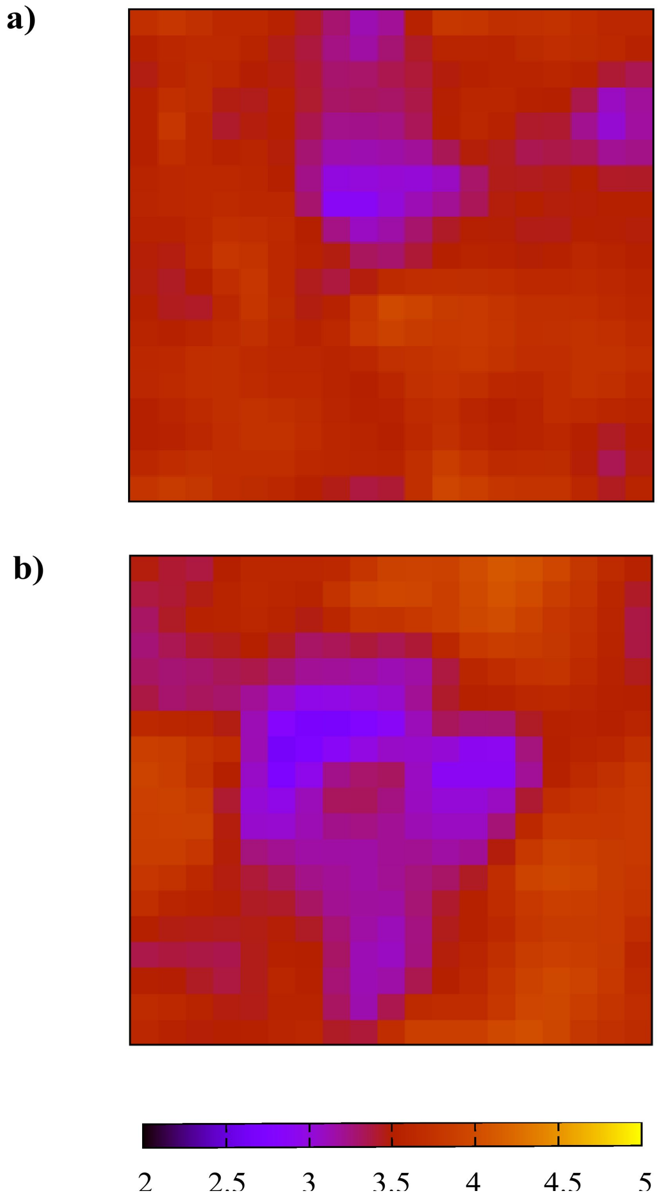

Figure S8: Bilayer phosphate-to-phosphate thickness, averaged over the last 400 ns of the 500 ns MD simulations of WT Aβ$_{1-42}$ as (a) β-sheet monomer and (b) β-sheet tetramer in a POPC bilayer. The axes of each plot corresponds to the *x*- and *y*- direction of the lipid bilayer (both about 6.5 nm). The bilayer thickness was calculated with GRIDMAT-MD using 20 grid points in both directions. In each case, the peptide (not shown) is located near the center of each square where the thickness is smallest. The legend shows bilayer thickness (nm), mapped to the corresponding colors.

Analysis of bilayer thickness for mutant $A\beta_{1-42}$ (monomer)

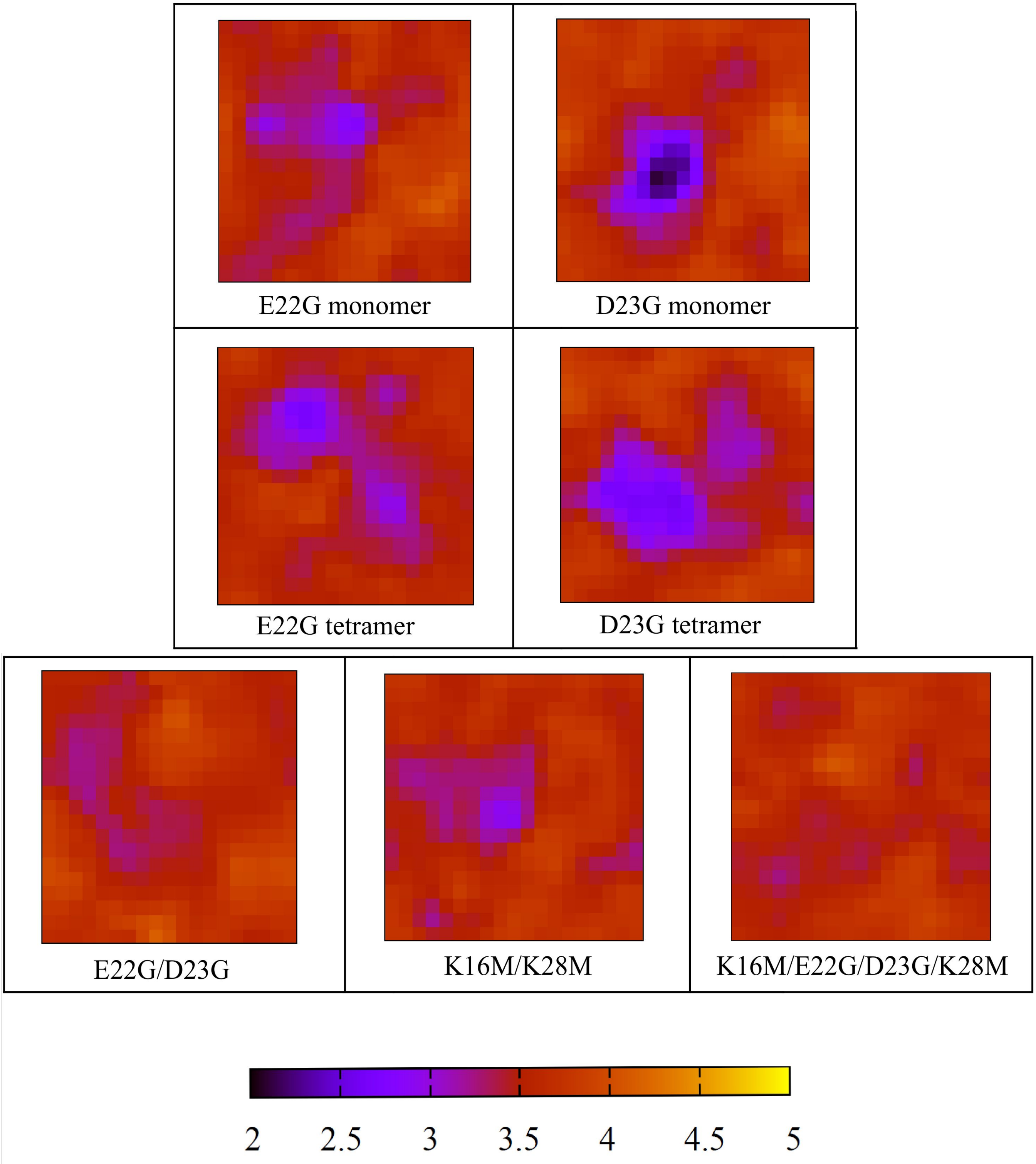

Figure S9: Bilayer phosphate-to-phosphate thickness, averaged over last 400 ns of the 500 ns MD simulations of $A\beta_{1-42}$ mutants (monomers and tetramers). For the coloring explanation, see Fig. S11.

Deuterium order parameters for A$\beta_{1\text{-}42}$ (monomer)–POPC bilayer systems

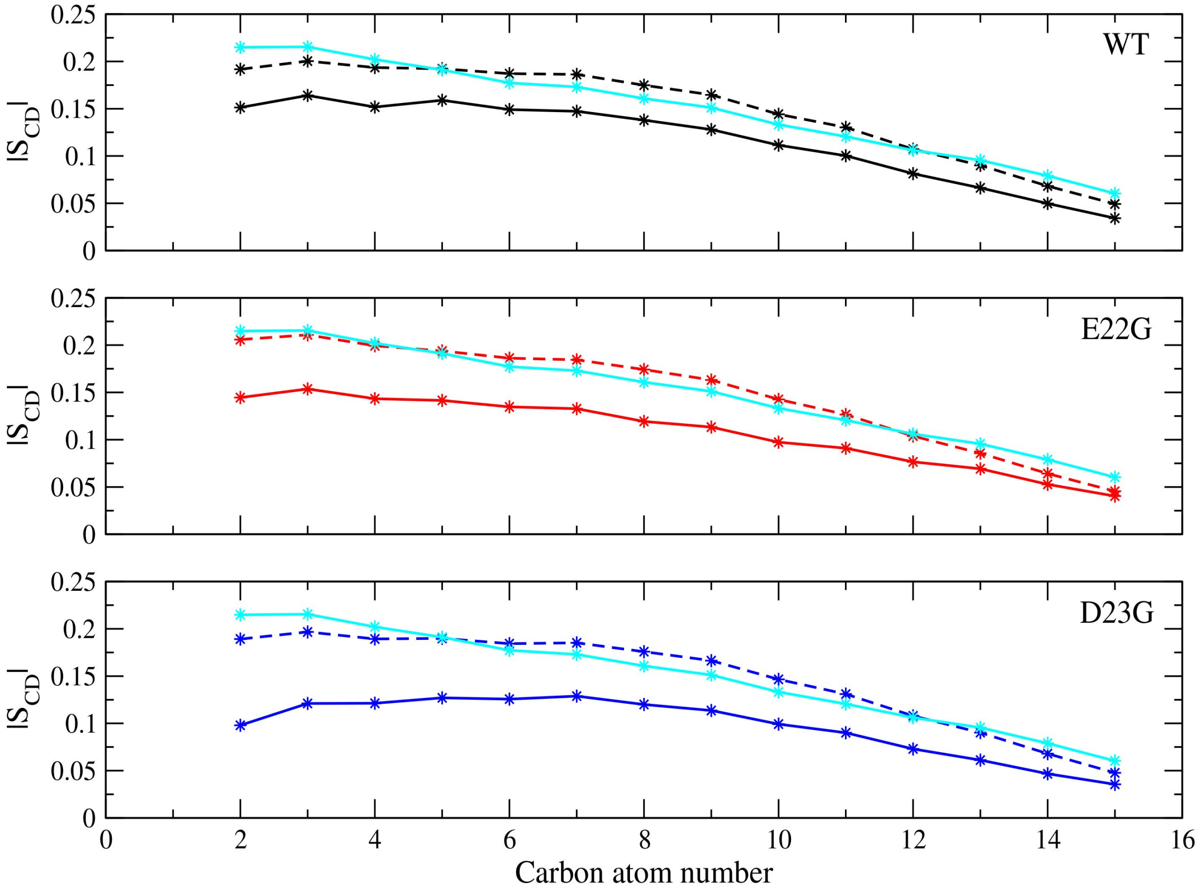

Figure S10: Time-averaged (over the last 400 ns of the MD simulations) order parameter S_{CD} of the palmitoyl chain of the POPC lipids. Results are shown for wild type and mutant A$\beta_{1\text{-}42}$ and are distinguished for the lipids within 5 Å of A$\beta_{1\text{-}42}$ (solid) and for the lipids >5 Å away from A$\beta_{1\text{-}42}$ (dashed). For comparison, S_{CD} of the palmitoyl chain obtained from a 100 ns MD run of peptide-free POPC bilayer is also presented (cyan).

Deuterium order parameters for A$\beta_{1\text{-}42}$ (monomer)–POPC bilayer systems

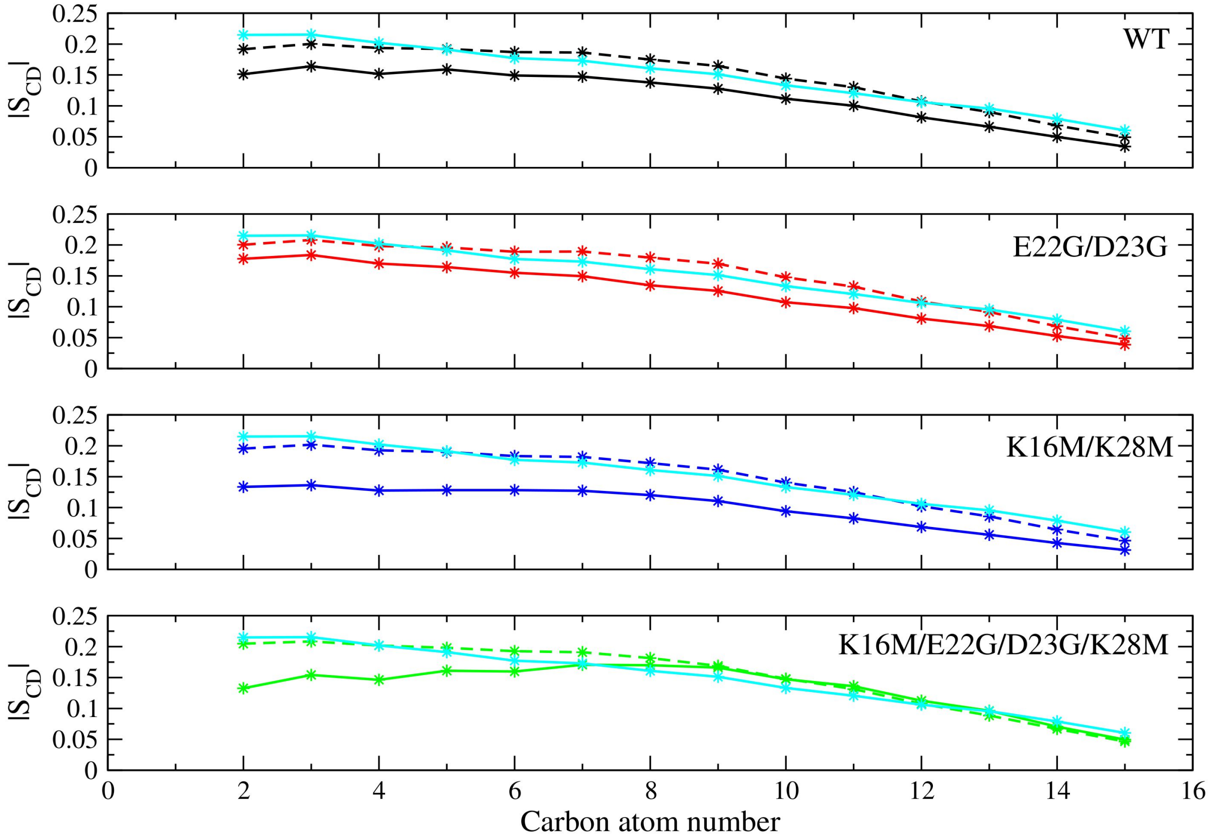

Figure S11: Time-averaged (over the last 400 ns of the MD simulations) order parameter S_{CD} of the palmitoyl chain of the POPC lipids. Results are shown for wild type and mutant A$\beta_{1\text{-}42}$ and are distinguished for the lipids within 5 Å of A$\beta_{1\text{-}42}$ (solid) and for the lipids >5 Å away from A$\beta_{1\text{-}42}$ (dashed). For comparison, S_{CD} of the palmitoyl chain obtained from a 100 ns MD run of peptide-free POPC bilayer is also presented (cyan).

Deuterium order parameters for Aβ$_{1-42}$ (tetramer)–POPC bilayer systems

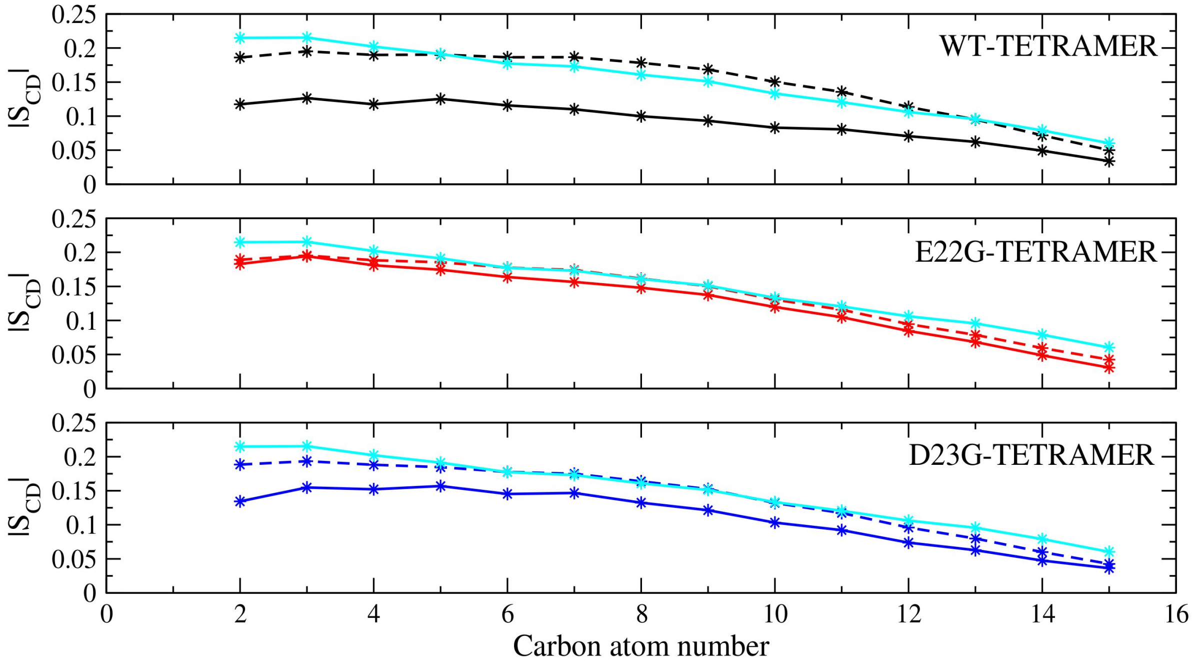

Figure S12: Time-averaged (over the last 400 ns of the MD simulations) order parameter S_{CD} of the palmitoyl chain of the POPC lipids. Results are shown for wild type and mutant Aβ$_{1-42}$ and are distinguished for the lipids within 5 Å of Aβ$_{1-42}$ (solid) and for the lipids >5 Å away from Aβ$_{1-42}$ (dashed). For comparison, S_{CD} of the palmitoyl chain obtained from a 100 ns MD run of peptide-free POPC bilayer is also presented (cyan).
